# Differences in Clinicopathology of Early Gastric Carcinoma between Proximal and Distal Location in 438 Chinese Patients

**DOI:** 10.1038/srep13439

**Published:** 2015-08-27

**Authors:** Qin Huang, Cheng Fang, Jiong Shi, Qi Sun, Hongyan Wu, Jason S. Gold, H. Christian Weber, Wenyan Guan, Yifen Zhang, Chenggong Yu, Xiaoping Zou, Hiroshi Mashimo

**Affiliations:** 1 Department of Pathology, the Nanjing Drum Tower Hospital, No. 321 Zhongshan Road, Nanjing 0210008, Jiangsu Province, P.R.China; 2 Department of Pathology and laboratory medicine, Veterans Affairs Boston Healthcare System and Harvard Medical School, 1400 VFW Parkway, West Roxbury, MA 02132, USA; 3 Department of Gastroenterology, the Affiliated Drum Tower Clinical Medical School, Nanjing Medical University, No. 321 Zhongshan Road, Nanjing, 0210008, Jiangsu Province, P.R.China; 4 Department of Surgery, Veterans Affairs Boston Healthcare System and Harvard Medical School, 1400 VFW Parkway, West Roxbury, MA 02132, USA; 5 Department of Gastroenterology, Veterans Affairs Boston Healthcare System and Harvard Medical School, 1400 VFW Parkway, West Roxbury, MA 02132, USA

## Abstract

Early gastric carcinoma (EGC) in Chinese patients remains poorly understood and endoscopic therapy has not been well established. Here, we compared endoscopic and clinicopathologic features between early proximal gastric carcinoma (PGC, n = 131) and distal gastric carcinoma (DGC, n = 307) in consecutive 438 EGCs diagnosed with the WHO criteria. By endoscopy, PGCs showed protruding and elevated patterns in 61.9%, while depressed and excavated patterns in 33.6%, which were significantly different from those (32.6% and 64.5%) in DGCs. PGCs were significantly smaller (1.9 cm in average, versus 2.2 cm in DGCs), invaded deeper (22.9% into SM2, versus 13% in DGCs), but had fewer (2.9%, versus 16.7% in DGCs) lymph node metastases. Papillary adenocarcinoma was significantly more frequent (32.1%, versus 12.1% in DGCs), as were mucinous and neuroendocrine carcinomas, carcinoma with lymphoid stroma (6.9%, versus 1.6% in DGCs); but poorly cohesive carcinoma was significantly less frequent (5.3%, versus 35.8% in DGCs). The overall 5-year survival rate was 92.9% in EGCs, and PGC patients showed shorter (42.4 months, versus 48.3 in DGCs) survival. Papillary and micropapillary adenocarcinomas and nodal metastasis were independent risk factors for worse survival in EGCs. EGCs in Chinese were heterogeneous with significant differences in endoscopy and clinicopathology between PGC and DGC.

Early gastric carcinoma (EGC) is defined by the 2010 World Health Organization (WHO) Classification of Tumours of the Digestive System as an invasive neoplasm confined to gastric mucosa or the submucosa, irrespective of the status of lymph node metastasis^1^. The importance of early detection of EGC with effective endoscopic resection has been demonstrated in Japan by excellent 5-year survival rates of about 90% or more^2^, compared to 14–25% for advanced gastric cancer^3^. Implementation of a population-based endoscopic screening program has been attributed to earlier detection and endoscopic resection of EGCs in Japan. As a result, the overall 5-year survival rates of gastric cancer patients are much higher (65–74%) in Japan than in other countries (10–30%)^4^. At present, the reported incidence of EGC in Western countries remains low (5–21%)^5^,^6^. Apart from the differences in genetic vulnerability and environmental factors among various populations, use of different histopathologic diagnostic criteria for EGC is believed to be one of major factors for the discrepancy in EGC incidence and survival between Japan and other countries^7^,^8^.

As in Japan, gastric cancer in China also has high prevalence and accounts for about half of all gastric cancer cases in the world^9^. In China, gastric cancer ranks as the 3^rd^ leading cancer incidence and the 2^nd^ most common cause of cancer-related deaths^10^,^11^. Although esophagogastroduodenoscopy has been widely available to citizens in China, detailed clinicopathologic characteristics of EGC remain lacking and therapeutic options are limited for this heterogeneous cancer. Compared to distal gastric carcinoma (DGC), proximal gastric carcinoma (PGC) in Chinese patients manifests predominance in the elderly, heterogeneous histopathology, and high expression of HER2 and Sirt1 genes^12^,^13^,^14^,^15^,^16^. However, differences in clinicopathology of EGCs between PGCs and DGCs are unknown. Therefore, in this study we applied the latest WHO diagnostic criteria to systemically investigate EGC and compare endoscopic and clinicopathologic characteristics between PGC and DGC in Chinese patients treated at a single high-volume tertiary medical center in China.

## Results

Among 3176 consecutive resections of gastric carcinoma (3097 surgical gastrectomies and 79 endoscopic resections), 438 (13.8%) were eligible for the study (361 by surgery, 59 by endoscopic resection, and 18 by both), in which 131 (30%) were classified as PGCs and 307 (70%) as DGCs. The average number of tumor-bearing histology slides reviewed per case was 3.2 (range: 1–12).

### Demographic Characteristics

Overall, the average age of patients was 60.5 years (range: 17–86) and the male-to-female patient ratio was 2.2. Compared to DGC patients (Table 1), PGC patients were significantly older (average: 64.2 years, range: 42–82, *p* < 0.001), and none were younger than 40, which was significantly different from DGC patients. Compared to DGCs, the male-to-female patient ratio was higher in PGCs, but the difference was not statistically significant (Table 1).

### Endoscopic Gross Features

**Figure 1:**
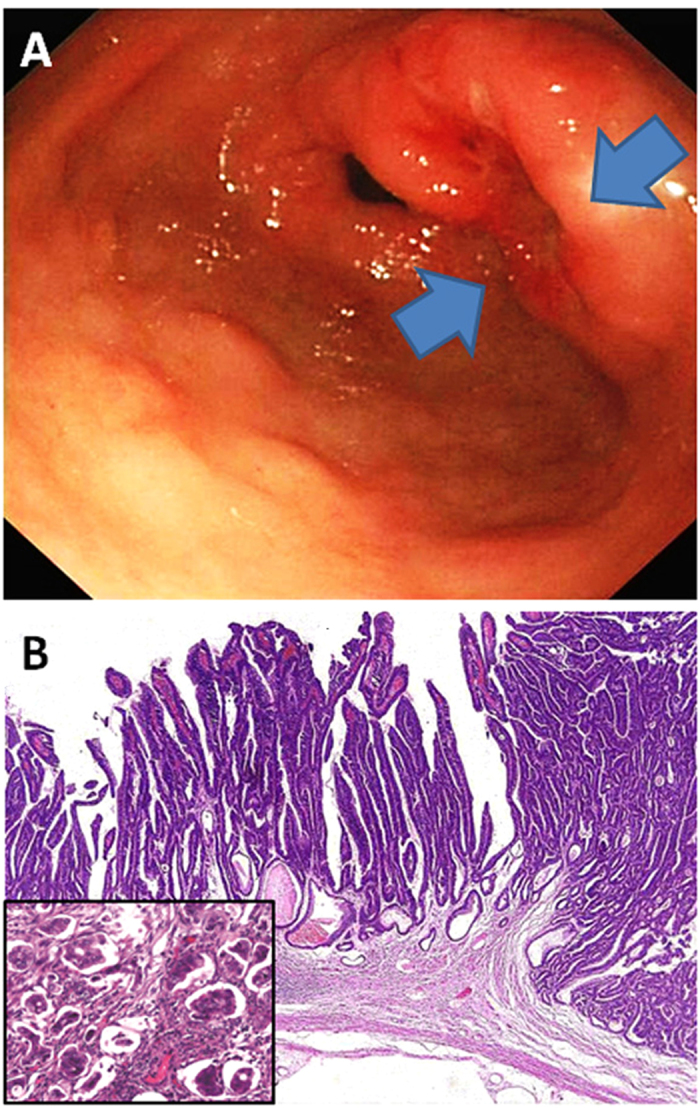
Representative antral papillary adenocarcinoma exhibiting an excavated gross appearance with defined borders (**A**), villiform papillary histology configuration (**B**), and a minor micropapillary component (insert).

**Figure 2:**
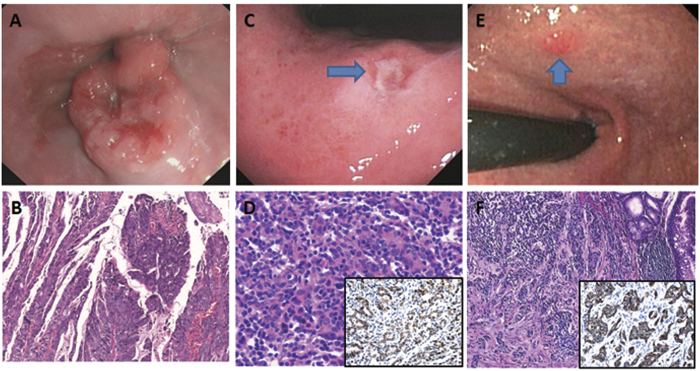
Uncommon carcinoma types in the proximal stomach exhibiting a protruded endoscopic pattern at the gastroesophageal junction (**A**) with a papillary histology type (**B**), or an excavated endoscopic appearance (**C**, arrow) and histology of carcinoma with lymphoid stroma (**D**), confirmed with positive *in situ* hybridization for the Epstein-Barr virus (insert), or a slightly elevated gross pattern (**E**, arrow) with neuroendocrine carcinoma histology (**F**), confirmed with positive immunostain for synaptophysin (insert).

**Figure 3:**
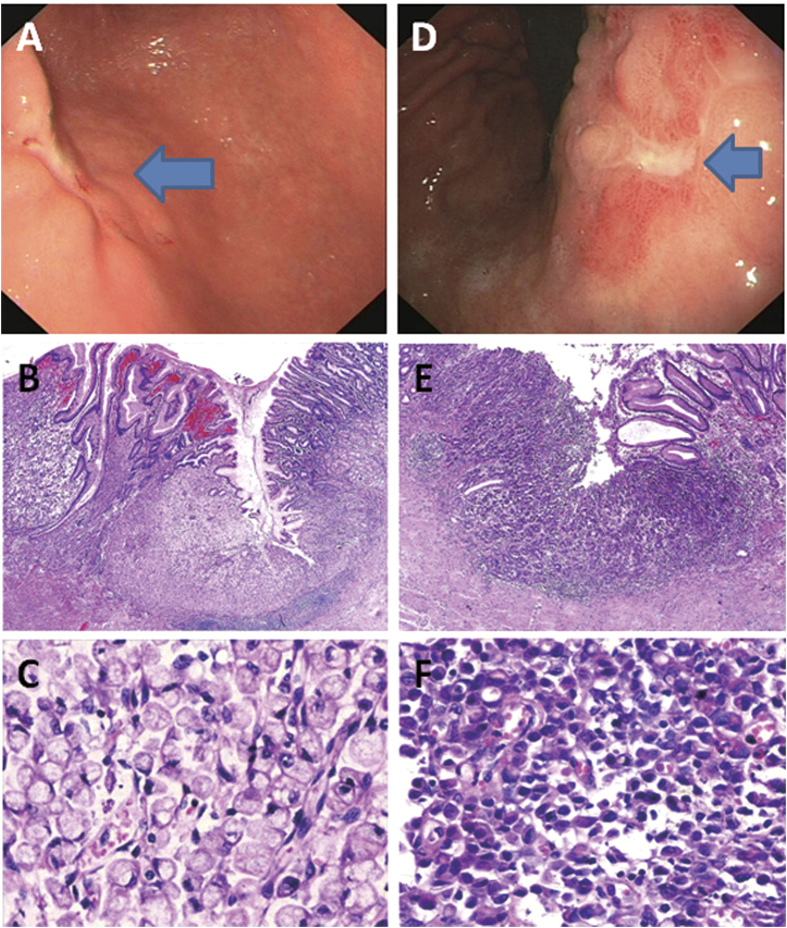
Two poorly cohesive carcinomas demonstrating a depressed endoscopic gross pattern (**A**, arrow) and signet-ring histomorphology in the corpus (**B**,**C**) and an excavated gross appearance (**D**, arrow), and poorly cohesive histology (**E**,**F**) in the body near the proximal stomach.

By conventional white light endoscopy, the most common mucosal pattern of EGCs was, in the descending order (Table 1), excavated (33.8%) (Figs 1A, 2C and 3D), elevated with rough surface (26.9%) (Fig. 2E), depressed with erosion (21.5%) (Fig. 3A), protruding (14.4%) (Fig. 2A), and flat (3.4%). Compared to DGCs, PGCs demonstrated significantly more frequent protruding and elevated patterns (61.9%), but fewer excavated and depressed patterns (33.6%) (*p* < 0.001). The average tumor size was significantly smaller in PGCs (1.9 cm, range: 0.3–5.5) than in DGCs (2.2 cm, range: 0.3–6.0) (*p* < 0.05). Interestingly, all 3 PGCs with nodal metastasis were smaller than 2 cm in size. One of these 3 PGCs had the protruding pattern and the other two were excavated. In contrast, 46 DGCs with nodal metastasis were significantly larger (average 2.6 cm, range: 0.5–6.0) and exhibited a predominantly excavated pattern (67%) (*p* < 0.001).

### Histopathology

Compared to DGCs (Table 1), PGCs invaded deeper with a higher frequency of submucosal invasion (52.7% versus 42.7% in DGCs). Most PGCs were significantly better differentiated; 59.5% were well-differentiated and only 16.8% were poorly differentiated (*p* < 0.001) (Table 1). While the proportion of tubular adenocarcinoma was similar between the two groups, papillary adenocarcinoma was significantly more common in PGCs (32.1%, Fig. 3B) than in DGCs (12.1%, Fig. 1B) (*p* < 0.001). All 4 micropapillary adenocarcinomas (1 PGC and 3 DGCs) were associated with a predominant papillary component (Fig. 1). Uncommon mucinous carcinoma, carcinoma with lymphoid stroma (only 1 PGC case; Fig. 2C,D), and neuroendocrine (Fig. 2E,F) carcinoma were also significantly more frequent in PGCs (6.9%) than in DGCs (1.6%) (*p* < 0.001). In contrast, poorly cohesive (including signet-ring cell) carcinoma was significantly less frequent in PGCs (5.3%) than in DGCs (35.8%) (Fig. 3, *p* < 0.001). Of 3 PGCs with nodal metastasis, two were poorly cohesive carcinomas and one was pancreatic acinar-like adenocarcinoma. The frequency of perineural and lymphovascular invasion was similar between the two groups (Table 1). However, the *H. pylori* infection rate (51.1%) in PGC was significantly lower than that (76.9%) in DGC (*p* < 0.001) (Table 1).

### Nodal Metastasis and Pathologic Staging

Nodal metastasis was evaluated in 379 (86.5%) cases with nodal dissection. The average number of lymph nodes retrieved per case was 18.8 and nodal metastasis was detected in 49 EGCs (12.9%). Nodal metastasis was significantly less frequent in intramucosal carcinomas (12/190, 6.3%) than in submucosal carcinomas (37/189, 19.6%) (*p* < 0.001). Half of the cases with micropapillary adenocarcinoma had nodal metastasis. Surprisingly, the nodal metastasis rate was significantly lower in PGCs (3/104, 2.9%) than in DGCs (46/275, 16.7%) (*p* < 0.001).

As shown in Table 1, the vast majority (97.1%) of PGCs were staged as pIA, while pIB was rare (2.9%). EGCs staged as pII were found only in the DGC group. Thus, the overall difference in staging between the two groups was significant (*p* < 0.001).

### Post-resection Survival

Forty-nine (11.2%) patients were lost to follow-ups (14 in the PGC group and 35 in the DGC). The median number of follow-up months after resection was 51 (range: 11–107). The overall average 5-year survival rate was 92.9% (Table 1). Compared to DGC patients, PGC patients showed a significantly shorter overall survival (42.4 months, versus 48.3 months in DGCs) (*p* < 0.05).

Univariate analysis revealed several significant risk factors for worse survival, including macroscopic protruded pattern (*p* < 0.05), histologic papillary (*p* < 0.05) and micropapillary (*p* < 0.05) types, nodal metastasis (*p* < 0.01), and summary pathology stage (*p* < 0.05) (Table 2). For the PGC group, none of the risk factors analyzed were statistically significant for survival prediction. In contrast, in the DGC group, the average tumor size larger than 2.1 cm (*p* < 0.05), macroscopic protruded pattern (*p* < 0.01), histologic papillary (*p* < 0.01) and micropapillary (*p* < 0.001) types, submucosal invasion (*p* < 0.05), nodal metastasis (*p* < 0.05), and summary pathologic stage (*p* < 0.05) were significant for predicting worse survival. Further multivariate analysis in all EGCs revealed that patients with papillary and micropapillary adenocarcinomas and those with nodal metastasis had worse survival (*p* < 0.05) (Table 3). In the DGC group, independent risk factors included both histologic papillary and micropapillary types (*p* < 0.05).

## Discussion

In this study, the proportion of EGCs in all gastric cancer resections at our hospital was 13.8%, comparable to that reported in Western countries^17^,^18^,^19^, but lower than that (>50%) in Japan^2^,^20^, We demonstrate that Chinese patients with EGCs diagnosed with the WHO criteria do have much better 5-year survival rates of over 92% after resection, which is similar to those reported in European and Japanese patients^17^,^20^. This excellent prognosis does not result from an over-diagnosis of EGC by including cases lacking invasive carcinoma^7^,^8^. Our data indicate a heterogeneous nature of EGC that can be divided into PGC and DGC subgroups. PGCs have distinct endoscopic and clinicopathologic features as follows: 1) PGC patients were mainly elderly and none were younger than 40 years; 2) most PGCs were small, protruding or elevated; 3) while adenocarcinoma remained predominant in PGCs, unusual histologic types, such as mucinous carcinoma, carcinoma with lymphoid stroma, and neuroendocrine carcinoma were also prevalent, but poorly cohesive (including signet-ring cell) carcinoma was significantly less frequent in PGCs; 4) PGCs were more deeply invasive but with fewer nodal metastases; 5) although *H. pylori* infection remained prevalent (69.4% for the cohort), the infection rate was significantly lower in PGC than in DGC, suggesting different pathogenesis mechanisms between PGC and DGC; and 6) despite the fact that while the vast majority (97.1%) of PGCs were staged as pIA, the overall survival was shorter and none of the known survival-related risk factors were found significant. The implications of these findings are at least three-fold. First, because of smaller size with fewer nodal metastases in most cases, early PGCs may be more suitable than DGCs for endoscopic resection. Second, the current pathology staging system cannot accurately stratify post-resection prognosis in PGC patients^21^,^22^. Given the rising incidence of PGCs in China^23^, a better understanding of risk factors in PGCs becomes critically important for disease prevention and development of a separate pathologic staging system for PGCs to guide patient management^21^,^22^. Finally, PGC appears to possess discrete gastric cancer pathobiology and differs from DGC in many aspects, supporting the proposed classification of gastric cancer into PGC and DGC subgroups^13^,^24^.

Nodal metastasis in EGC has been repeatedly confirmed as the most important independent risk factor for survival and as a relative contraindication for endoscopic resection^18^,^19^,^20^. This is also our experience. In this study, the overall nodal metastasis rate was 12.9% (6.3% for intra-mucosal and 19.6% for submucosal carcinomas), similar to that reported in another Chinese (12.2%)^25^, Korean (11.8%)^26^, and German (11.8%)^27^, Italian (14.1%)^18^, and other European studies^17^. This rate of nodal metastasis may reflect the appropriate frequency of nodal disease in early gastric carcinoma diagnosed with the WHO criteria in different populations. However, the reported nodal metastasis rate is much lower in Japanese series^20^,^28^. Tsujitani *et al.* described nodal metastasis rates of 1.1% for intra-mucosal and 15.8% for submucosal carcinomas^28^, which provided the basis for the Japanese treatment guidelines for EGC^20^. Such lower rates reported in Japanese patients may have resulted from inclusion of the cases without invasive carcinoma because invasion is not used as an essential criterion for EGC diagnosis in that country^7^,^8^. In 18 patients of this cohort with positive endoscopic resection margins and/or the fear of nodal metastasis, additional surgical resections with nodal dissection were carried out but revealed no positive lymph nodes in surgical specimens. Further investigation for risk factors of nodal metastasis in EGC in Chinese patients is needed to guide future patient management.

An interesting, but unexpected finding is the identification of papillary and micropapillary adenocarcinomas as independent risk factors for worse prognosis in EGC patients. Although the mechanisms for this finding remain unclear, EGCs with papillary adenocarcinoma have been shown to have frequent lymph node and liver metastases, worse overall 5-year survival^29^. This cancer is frequently associated with the recently established micropapillary adenocarcinoma of the stomach for propensity of nodal metastasis,^12^,^30^ as confirmed in the current cohort. However, contrary to the finding of worse survival in our EGC patients with this carcinoma, Roh *et al.* did not detect statistically significant differences in survival between micropapillary and control gastric carcinomas^30^. This discrepancy appears to result from more advanced gastric carcinomas investigated in that report with a median survival of only 18 months in the control group. Intriguingly, although poorly cohesive carcinoma (including signet-ring cell carcinoma) is well recognized for fatal outcomes in advanced gastric cancer, classified as undifferentiated carcinoma, and deemed unsuitable for endoscopic resection in EGC^5^,^20^, our data suggest that this cancer could be cured when discovered at an early stage, despite the more frequent submucosal invasion. In fact, EGCs with poorly cohesive carcinoma (including signet-ring cell carcinoma) have been shown not only to have a better prognosis than those of non-poorly cohesive carcinoma (including non-signet-ring cell carcinomas)^31^, but also to be more suitable for endoscopic resection^32^, which is consistent with our findings.

PGCs are common in the Chinese population and account for about one third of all gastric cancer resections, as shown in our current and previous studies^13^,^23^. This cancer has been considered as part of the EAC spectrum by Western investigators, based on the assumption that PGCs may arise from short-segment Barrett’s esophagus^33^. This notion is at odds with the findings in Chinese patients^12^, in whom both Barrett’s esophagus and EAC currently remain rare^34^. In a previous pathology study, we investigated histopathology of consecutive 204 qualified distal esophageal carcinoma resections performed at our center over the 7-year period. We found that EAC accounted for only 1% of all carcinomas^35^. Even in the region in China with the highest esophageal cancer incidence in the world, EAC stays scarce^36^. Interestingly, EAC in Hong Kong is not only consistently uncommon but also decreasing in incidence over the past decades^37^. In this study, PGCs were associated with advanced age but not with male gender, and showed different endoscopic gross and microscopic heterogeneous features, and unfavorable prognosis. Thus, such clinical, endoscopic, and pathologic characteristics of PGCs in the Chinese population are dissimilar, in most part, to those of EACs^12^,^34^.

The major limitation of this investigation is the retrospective study design. Although pre-operative upper endoscopy with biopsy before resection was routinely carried out, digital endoscopic and surgical resection specimen gross images were not available for review in all cases. Moreover, 11.2% of cases were lost to follow-up for survival analysis. However, the current cohort used the latest WHO diagnostic criteria on EGC with 438 resection cases, including over 130 consecutive early PGCs, which is exceptional, even in the published Japanese studies^5^,^20^,^28^,^29^.

In conclusion, EGCs diagnosed with the WHO criteria in Chinese patients is heterogeneous and can be divided into PGC and DGC subgroups. Compared to DGCs, early PGCs feature smaller size, deeper invasion, but fewer nodal metastases, and thus more suitable for endoscopic resection.

## Methods

### Case Selection

**Figure 4:**
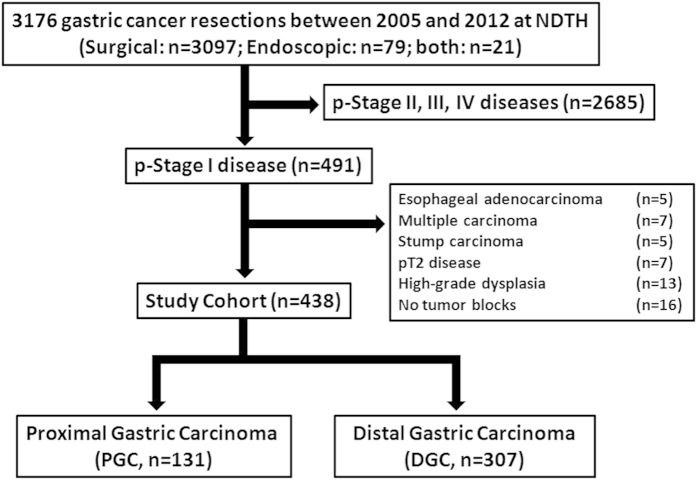
Flowchart of study case selections. Among 3176 surgical and/or endoscopic resections of gastric cancer, 491 were identified with pathological stage I diseases. After review of histology slides and reports, 53 were excluded for a variety of reasons, resulting in 438 cases that were further divided into proximal (PGC) and distal (DGC) gastric carcinoma groups. NDTH: Nanjing Drum Tower Hospital.

Consecutive surgical and endoscopic resection cases with a final pathologic diagnosis of gastric carcinoma were searched in the electronic pathology databank stored in the Department of Pathology of the Nanjing Drum Tower Hospital in China for the period from January 2005 to December 2012. Each pathology report was investigated for the depth of tumor invasion. Included in the study cohort were cases with invasion of neoplastic glands and/or cells into the lamina propria, muscularis mucosa (pT1a), or the submucosal space (pT1b), as defined by the 2010 WHO classification for EGC^1^. All histology slides were reviewed again by two pathologists for verification of the EGC diagnosis and the tumor invasion depth. Excluded were cases with: 1) no definitive evidence of invasion, 2) invasion beyond the submucosa, 3) synchronous tumors with a distance of at least 2 cm in between, 4) stump gastric carcinoma, 5) a history of prior neoadjuvant therapy, and 6) no tumor tissue blocks for recut (Fig. 4). The information on demographics and endoscopic/gross tumor characteristics was gleaned from patient medical records. All patients were followed up to confirm survival status by telephone/personal interview of the patient or family members. Patient consent for surgery and research was obtained prior to the resection procedure, which was carried out in accordance with the approved guidelines. The study protocol was approved by the Medical Ethics Committee of the Nanjing Drum Tower Hospital.

### Histopathology

Endoscopic and surgical resection specimens were routinely processed by a standard protocol^12^. All specimens were fixed in 10% buffered formalin solution overnight. All resection margins were inked. Gross characteristics of tumors, including size, shape, surface, color, and consistency were assessed along with endoscopic tumor images and reports to ensure data accuracy. Endoscopic tumor macroscopic appearances were classified into 5 patterns: 1) protruding, 2) elevated with a rough surface, 3) flat, 4) depressed with eroded surface, and 5) excavated^1^. By location, tumors were divided into two groups: 1) PGCs, defined as tumors with epicenter located about 3 cm distal to the gastroesophageal junction^13^,^21^ and 2) DGCs, tumors arising from all other regions of the stomach.

According to the 2010 WHO classification of gastric cancer, all EGC tumors were categorized into 6 major histopathologic types as adenocarcinoma, adenosquamous, mucinous, poorly cohesive (including signet-ring cell), and neuroendocrine carcinomas, and carcinoma with lymphoid stroma. Micropapillary adenocarcinoma was defined as small pseudopapillary tumor clusters in at least 5% of the estimated tumor volume without fibrovascular cores but surrounded by empty lacuna spaces^30^. Pancreatic acinar-like adenocarcinoma was determined by the criteria described previously^38^. All tumors were graded for differentiation, according to the WHO criteria^1^. Well-differentiated tumors showed well-formed tubules or papillae in over 95% of the estimated tumor mass, while the poorly differentiated exhibited irregular, indiscernible glands in less than 50%. Also recorded were perineural and lymphovascular invasion, and the status of the resection margin. For cases in which initial tumor sampling failed to show invasive carcinoma, the entire gastric mucosa was subjected to microscopic evaluation. In tumors showing histologic impression of neuroendocrine carcinoma or carcinoma with lymphoid stroma, an immunohistochemical or *in situ* hybridization test was carried out on the same tumor block for verification.

Also tabulated was pathology discovered in the uninvolved gastric mucosa such as chronic active gastritis, *H. pylori* infection (identified on H&E or Giemsa stain), metaplasia (intestinal and pancreatic), atrophy (defined as reduction in the number of gastric glands or the presence of intestinal metaplasia), and gastritis cystica profunda (defined as benign ectatic gastric glands in the submucosal space)

Lymph node metastasis was investigated in cases with open surgical nodal dissection. Pathologic staging was based on the 7^th^ edition of the American Joint Committee on Cancer (AJCC) staging manual^39^.

### Immunohistochemistry

Immunohistochemical studies were performed using conventional methods^12^,^13^. Appropriate positive and negative controls were included in each run to safeguard the test validity. For neuroendocrine carcinoma, anti-synaptophysin antibody was used (clone 27G12, dilution 1:250, Novocastra, the United Kingdom). For pancreatic acinar-like adenocarcinoma, anti-α1-chymotrypsin antibody was utilized (polyclonal, dilution 1:100, Zymed Labs, CA). Immunoreactivity was considered positive for pancreatic acinar-like adenocarcinoma if over 10% of total target cells were stained on the same tissue section^12^,^38^.

Each immunostained slide (along with each routine histology case) was reviewed independently by two experienced pathologists blinded to the clinicopathologic and survival information. The differences were minimal and resolved with consensus.

### In Situ Hybridization for Epstein-Barr Virus (EBV)-encoded Small Ribonucleic Acid-1

Tumors with dense small lymphocytic infiltrate on routine histology sections were selected for the EBV *in situ* hybridization test, as described previously^13^,^40^. Tissue sections were sequentially deparaffinized, rehydrated through graded ethanol solutions in a descending order down to water, predigested with 0.4% peptidase, and hybridized overnight at 37 °C with digoxigenin-labeled probes, according to the manufacturer’s instruction (Zhongshan Jingqiao, Beijing, China). After washing with phosphate buffer saline solution, the hybridization signal was detected using an anti-digoxigenin antibody-horseradish peroxidase conjugate and counterstained with hematoxylin. The positive control consisted of Burkitt’s lymphoma and a normal lymph node served as the negative control. Both controls were run in each batch to ensure test validity.

### Statistical Analysis

Numerical, continuous, and categorical variables were statistically analyzed for differences between groups in age, gender, tumor location, size, endoscopic appearance, type, differentiation, perineural and lymphovascular invasion, pathologic stage, tumor recurrence, and post-operative survival. The Chi-square, Fisher’s exact, or Kruskal-Wallis H test was employed, when appropriate. The patient post-resection survival period was calculated from the month of resection to the month of the last follow-up or death of all causes. The survival data were censored for patients who were alive at the last follow-up and analyzed with one-way ANOVA. The Statistical Package for Social Sciences (SPSS, version 13, Chicago, USA) was utilized for all statistical analyses. *P* values < 0.05 were defined as statistically significant.

## Additional Information

**How to cite this article**: Huang, Q. *et al.* Differences in Clinicopathology of Early Gastric Carcinoma between Proximal and Distal Location in 438 Chinese Patients. *Sci. Rep.* **5**, 13439; doi: 10.1038/srep13439 (2015).

## Figures and Tables

**Table 1:**
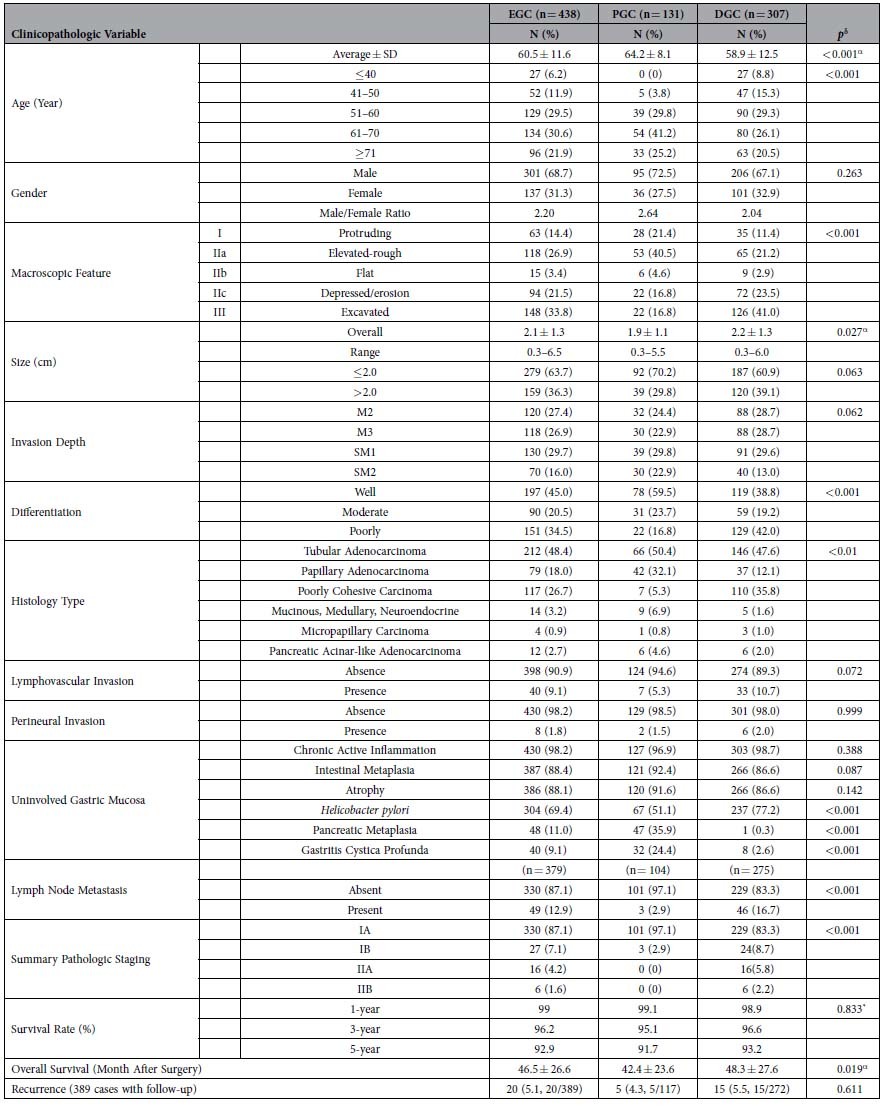
Comparison of Clinicopathologic Features between Early Proximal and Distal Gastric Carcinomas.

EGC: early gastric carcinoma; PGC: proximal gastric carcinoma; DGC: distal gastric carcinoma; SD: Standard Deviation; α: One-way ANOVA test; *: Log-rank test; δ: Chi-Square Test.

**Table 2:**
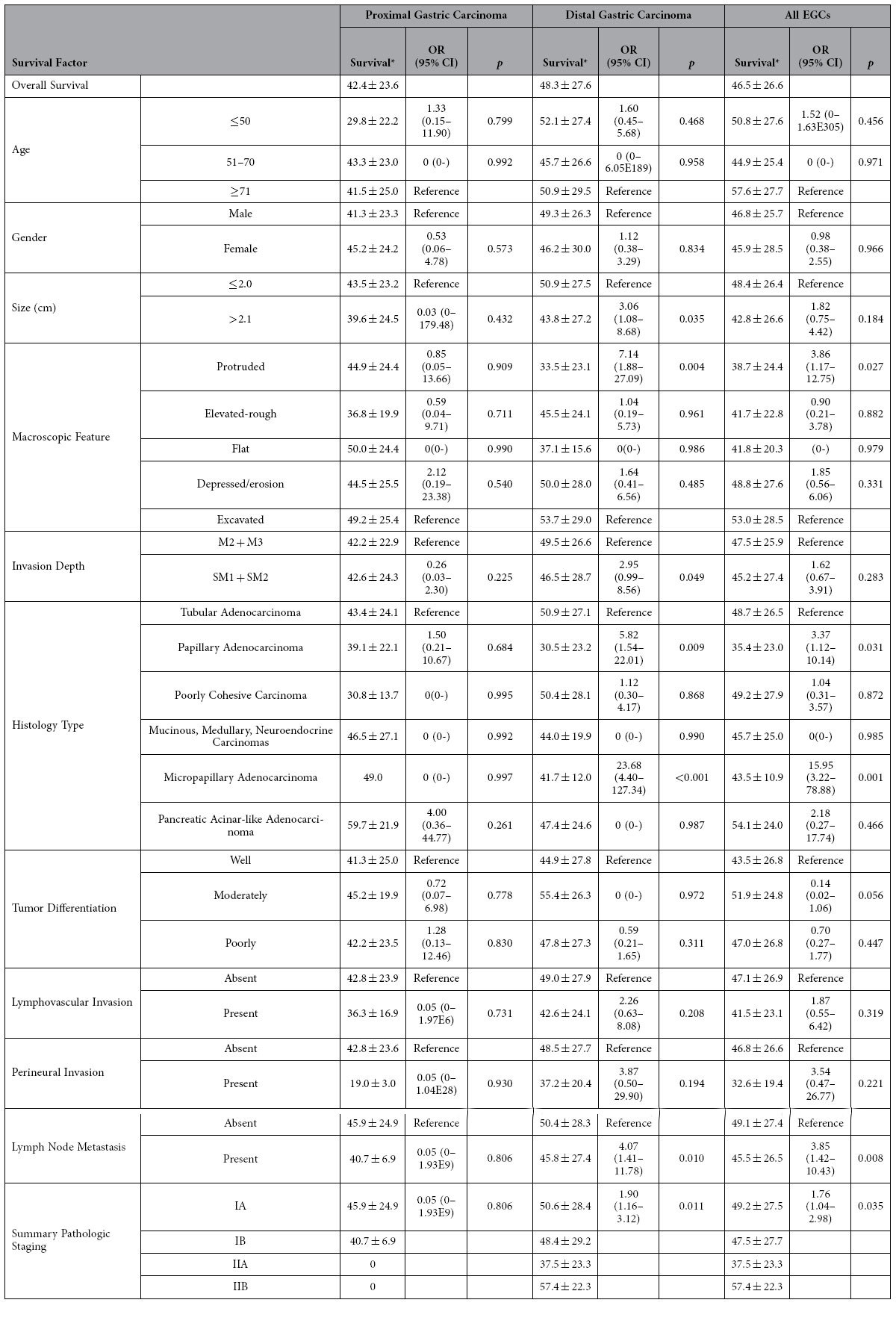
Univariate Analysis of Relationships between Clinical, Endoscopic, Pathologic characteristics, and post-operative survival.

*: Number of months after resection, average ± Standard Deviation; EGC: early gastric carcinoma; OR: Odds Ratio to increased risk for death; CI: confidence interval.

**Table 3:**
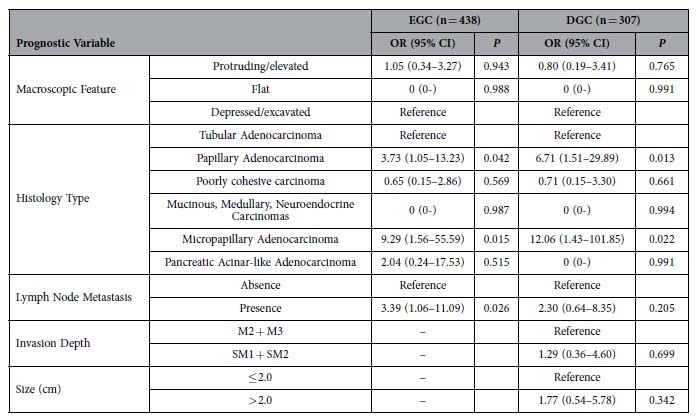
Multivariate Analysis of Prognostic Factors in Early and Distal Gastric Carcinomas.

EGC: early gastric carcinoma; DGC: distal gastric carcinoma; OR: Odds Ratio to increased risk for death; CI: confidence interval.
